# Solid organ transplantation in older adults. Infectious and other age-related considerations.

**DOI:** 10.21926/obm.transplant.1901046

**Published:** 2019-02-01

**Authors:** Marion Hemmersbach-Miller, Cameron R. Wolfe, Kenneth E. Schmader

**Affiliations:** 1. Division of Infectious Diseases, Duke University Medical Center, Durham NC, USA; 2. Duke Clinical Research Institute, Durham NC, USA; 3. Division of Geriatrics, Duke University Medical Center, Durham NC, USA; 4. GRECC, Durham VA, Durham NC. USA

**Keywords:** Aging, Infections, Immunocompromised Host

## Abstract

In the U.S., older adults aged 65 or above comprise nearly one quarter of the solid organ transplant (SOT) waitlists, and the number of transplants performed in this age group continues to increase. There are no specific guidelines for the assessment and follow up of the older SOT candidate or recipient. Older adults are at increased risk of infectious complications after SOT. Despite these complications and even with the use of suboptimal donors, overall outcomes are favorable. We provide an overview to specific consideration as they relate to the older SOT candidate and recipient.

## Introduction

**Figure 1.**
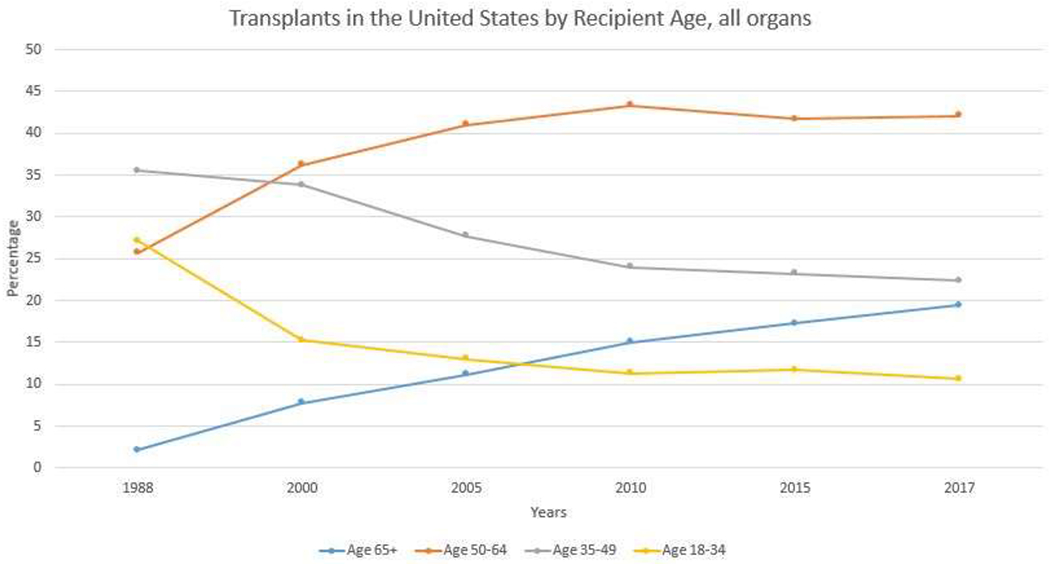
Transplants in the United States by Recipient Age, all organs. Based on OPTN data as of November 26, 2018.

Over 114,000 people are currently on the transplant waitlist; nearly a quarter of these (23.4%) are 65 years of age or older. Additionally, the number of transplant recipients in this age group has steadily increased over the last 20 years (Figure 1). There are no good estimates of people living with end-stage-organ disease in general as many are not transplant candidates for different reasons, but due to an increasing life expectancy[1] with subsequent end-stage-organ disease, it is only reasonable to assume that this number is increasing as well[2].

Age alone is no longer a contraindication for transplantation. In many centers, it has become the treatment of choice for end-stage-organ-disease for people deemed capable of tolerating the procedure. However, infectious complications threaten favorable outcomes in organ transplant recipients of any age, and this is exacerbated in older adults due to their higher risk of infections in general[3, 4]. There are many challenges and differences in epidemiology, pathogenesis, diagnostic approach and treatment of infections in older adults[3], organ transplantation adds another level of complexity. Older adults present for solid organ transplantation (SOT) as a result of a wider array of diseases and disorders resulting in a variety of infections compared with the general populations[5–7].

In this paper, we will provide an overview to specific consideration as they relate to the older, 65 years and above, SOT candidate and recipient.

## Special pre-transplant considerations for older recipients

Pre-transplant evaluations for older adults often vary somewhat from their younger counterparts. While center-specific standard evaluation protocols should be followed, there are several considerations related to the selection of older donors and recipients, as well as some particular terms that the reader should be familiar with. These special considerations are not only related to frailty and resilience, but also need to include assessing activities of daily living, physical and cognitive function, and goals of care. Older SOT candidates must be able to perform day-to-day tasks such as complex medication and dietary management, and have ability to attend follow up clinic visits even in the early post-transplant period. Social support systems must be in place and potential social barriers need to be identified.

A greater number of comorbidities typically occurs in older transplant candidates, e.g. diabetes mellitus, renal dysfunction, cardiovascular disease, hypertension, prior malignancy etc.[8–10]. In order for older adults to be evaluated for transplantation, their life expectancy should be longer than the *average* time on the waitlist. Certain comorbidities might be more or less impactful depending on the type of SOT, as such e.g. kidney transplant candidates might require abdominal imaging to look at the vascular bed[11] and testing for cardiovascular disease[12]. Societal and center-specific guidelines are followed for the different organ groups.

*Frailty* is generally defined as a syndrome of physiological decline in late life, characterized by vulnerability to adverse health outcomes. Its definition has slightly varied over the last decades and there have been numerous ways of evaluating frailty in a more standardized way[13, 14]. Distinguishing frailty from comorbidities, cognitive decline, impaired or lack of functionality or disability might become difficult[15]. Regardless of which scale or mechanism is used, it becomes clear that frailty is related to post-transplant outcomes[16] and operative mortality[17, 18]. A functional and cognitive assessment pre-transplant could improve post-transplant outcomes in this vulnerable population. This should also include screening for depression and underlying psychiatric illnesses. Comorbidities should be taken into account but some comorbidity indexes might not be accurate in the pre-transplant setting of end-stage-organ-disease(s)[19, 20]. Additionally, for example, gait speed may be difficult to assess in patient with end-stage heart or lung disease; weight loss may be hard to interpret for patients that are receiving diuretics or are on dialysis.

*Resilience* has been described as the capacity to maintain or regain well-being during or after adversity. Physical resilience[21] is a newer concept that might be applicable to older transplant recipients as well, yet there is no consensus in how to define or best measure it[22].

Assessing activities of daily living, physical and cognitive function should include objective measures. Self-reported functionality often differs from actual measured performance[23] which can be objectively evaluated using various tests or scales. Additionally, functional or cognitive decline can present earlier in the life, e.g. cognitive impairment in end-stage-renal-disease[24]. Our center requires older lung transplant candidates to remain out of the hospital to remain actively listed.

An additional important consideration in the United States is the patient transitioning to Medicare-based care at age 65. Given this transition, the increased recognition of cost and potential post-transplant medication coverage need to be pro-actively addressed in the pre-transplant setting. Medicare Part A covers organ transplants in certain conditions, and so does Medicare Part B for certain follow up visits, testing and lab work. Medicare Part D might become necessary for extended medication coverage. As in any patient, medication coverage needs to be assured to avoid potential post-transplant complications due to infections and/or rejection due to unaffordability of drugs. Recipients should understand and know what copays they might face.

Although end-of-life wishes and/or considerations should be addressed in any hospitalized patient, especially those about to undergo significant surgery with the potential of morbidity and mortality of SOT, it likely becomes even more important in this age group. A good understanding of the transplant process and potential physical and psychological burden that it can imply is imperative to minimize preventable difficulties.

Finally, there is an absence of alternative measurements for transplant success, and older adults might consider endpoints other than long-term survival as critically important (e.g. quality of life, independence). These are important considerations for transplant recipients of all ages but younger recipients might consider long-term survival as highly important. Measuring success is a major opportunity for future research.

### Deceased donor considerations

Increased recipient age generally allows for the use of a wider pool of donors, e.g. in the U.S. this might include older[25], expanded criteria and increased risk donors[26–28]. Certain organs from donors where graft survival and longevity might not be as long as classically from younger donors, e.g. high kidney donor profile index (KDPI) kidneys, might be safe to use in these older recipients[29, 30]. This has also led some programs to accept kidneys from hepatitis C (HCV) infected donors for older recipients, therefore expanding the donor pool and utilizing a kidney that otherwise would have been discarded[31].

## Organ specific considerations in older recipients. Peri- and post-transplant period.

Age itself is an independent risk factor for postoperative morbidity and mortality[32–39]. The fact that transplant surgery is often long, involves patients with several comorbidities, often results in high volume transfusions in certain cases, especially increases the perioperative infection risk [33, 40]. But even taking this into account, after about 3 months there is a survival benefit from transplant over dialysis in patients with end-stage-renal-disease [33, 41]. Short term outcomes in the peri-transplant period for other SOTs are less well studied in this age group[42], but e.g. in lung transplant recipients the increase mortality is mainly seen after the first three months of surgery[43].

A natural timeline of infectious complications in SOT recipients has been accepted for many years[44]. It suggests a dynamic assessment of infection risk over time and divides the post-transplant period in several time frames. During the first month after transplant, the main infections will be either donor derived or nosocomial related to the procedure or the hospital stay. An exception to this would be patients that are colonized prior to transplant, such as it often happens with certain lung transplant candidates, e.g. older adults presenting with complications of chronic bronchiectasis. The period between 1-6 months sees a variety of bacterial, viral or fungal infections that will largely depend on the type of prophylaxis used. After the initial 6 months, patients will present with any type of community-acquired infection and/or reactivation of latent infections such as of the herpesviridae group (mainly cytomegalovirus (CMV), HSV, VZV). Types of infection will also depend on the donors and recipients’ CMV serostatus as well as the prophylaxis and its duration that varies by organ and center.

Interestingly, while the transplant community has accepted this changing timeline, there are many distinctions that make specific infections in older adults potentially more frequent as this population is already at increased *baseline risk* e.g. for pneumonia, urinary tract infections (UTIs), Clostridium-difficile infections[3].

Knowledge of local prophylactic protocols, resistance patterns and allocations is therefore imperative when evaluating the aged SOT recipient.

### Heart transplantation

**Figure 2.**
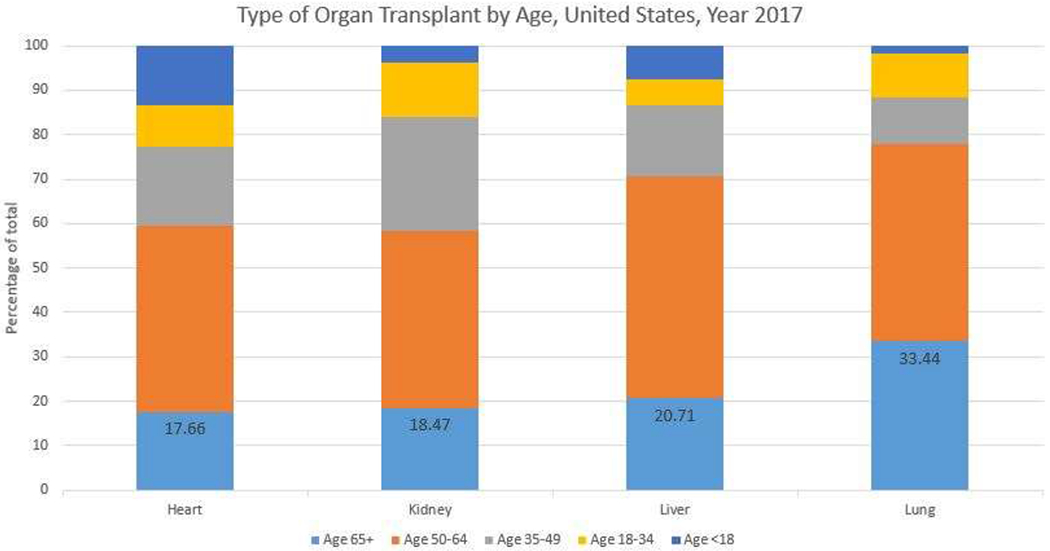
Type of Organ Transplant by Recipient Age, United States, Year 2017. Based on OPTN data as of November 26, 2018.

One of the most remarkable demographic trends in heart transplantation according to the latest SRTR/OPTN Annual Data Report is a steady increase in the proportion of heart transplant candidates aged 65 or older, with a growing proportion from 9.7% in 2005 to 17.4% in 2016[36]. Also see Figure 2. Survival at 5 years was 76.6% in this age group. Cardiomyopathy and coronary artery disease are the main reasons for end-stage-heart-disease. Particular to the heart transplants and due to the scarcity of organs overall, in older adults, end-stage-heart-disease has led to more destination left ventricular assist devices (VADs) which can provide a good alternative to transplant, with manageable symptoms yet without the risks of immunosuppression. VADs have also been associated with a risk of infection[45] and an increase in suppressive T-regulatory cells[46]. Overall, the distribution of adults on the waitlist has shifted to more patients being transplanted from VAD support[36], despite evidence that older patients have worse outcomes, and higher risk of stroke and gastrointestinal bleeding with VAD therapy[47].

Infection remains the number one reason for one-year cumulative incidence of death by cause among adult heart transplant recipients[36], and continuous over several decades after transplantation[48]. Non-CMV infections are predominant in the early period after transplantation[49]. Type of infections depend on the clinical scenario. For example, a patient that has a VAD as a bridge to transplantation with a potential history of driveline or pump infection(s) will have a different risk, typically with more bacterial complications than someone that requires extracorporeal membrane oxygenation versus someone with an uneventful peri-transplant course. These patients are at risk for early infections, especially bacterial pathogens from the driveline or pump pocket and Candida spp. Interestingly, older adults seem to have a much lower incidence of driveline infection when compared with younger patients, possibly a reflection of the younger patients being more ill at the time of VAD implant[47] or potentially more active and less compliant with dressing changes.

Another unique risk associated with heart transplants is their predisposition to toxoplasmosis or Chagas disease[50] as the allografts themselves can carry it. Clinical toxoplasmosis more frequently occurs following recent seroconversion[51], with the risk in this group of seronegative recipients being as high as 50-75% in the absence of antimicrobial prophylaxis[52]. Chagas disease can reactivate at any time after transplantation, primarily in the early post-transplant period and in instances where increased immunosuppression is needed[53]. There is limited data on the outcomes of disseminated toxoplasmosis or Chagas in older adults although drug tolerability might become an issue.

Finally, as a heart transplant candidate ages, they have a greater chance of cumulative exposure and infection with Tuberculosis, endemic fungi like *Coccidioides immitis* or helminths such as *Strongyloides stercoralis*[54–57]. A more detailed geographic history pre-transplant especially when weighing up whether the immunosuppressive reactivation of some of these devastating latent infections lends itself to believing that a VAD becomes a better option. Overall, identifying which older adults would be more appropriate for transplantation versus VAD therapy could help inform clinical decision-making in the future[30].

### Kidney transplantation

The proportion of older adults, that is aged 65 or above, on the kidney transplant waitlist continues to increase reaching over 20% at the time of the last Annual Report in 2016[38]. Data show that appropriately selected older adults with end-stage-renal-disease who undergo kidney transplantation have a survival benefit over those who remain on dialysis[58].

Infections imply a higher incidence of short- and long-term morbidity and mortality in this age group[5, 59, 60]. Infections are among the top three causes of death. Death with a functioning graft is by far the most common form of graft loss in older adults[61, 62]. Infections are also a very frequent reason of readmission in the first year after transplantation for the older recipient[60].

As for any post-transplant infection, types of infections vary depending on patient-specific risks. Patients that are on dialysis prior to transplantation do have different risks. Within this group, modes of dialysis portend different risks, e.g. hemodialysis versus peritoneal dialysis, hemodialysis via permcath, fistula or graft, history of prior peritonitis etc. Other factors are living donor availability, delayed graft function[63], use of ureteral stents[64], need for percutaneous nephrostomy tubes[65] etc.

Additionally, kidney transplantation can be unique as more patients than other transplant candidates will have a history of a prior transplantation and will already be immunosuppressed going into surgery, e.g. due to calcineurin inhibitor (CNI) toxicity[66, 67], or infectious complications during a previous transplant. As such, CNI induced nephrotoxicity has been described as being universally present at 10 years after kidney transplantation[68], and at least a contributory cause of renal toxicity after other types of SOTs[67].

Bacterial infections predominate in kidney transplant recipients, especially in older adults; the most common post-transplant infections are UTIs[6] followed by respiratory infections. Reactivation of herpesviridae, especially CMV are common[60] with timing and severity depending on the donor and recipients’ serostatus. Although not unique to kidney transplants, BK polyoma virus (BKV) viremia and nephropathy is often associated with rejection and can result in graft loss as well as increased comorbidities[69, 70]. Screening for BKV viremia has become standard of care in kidney transplant recipients[70], and it appears to be more frequent with advancing age[71].

### Liver transplantation

The proportion of liver transplant candidates aged 65 or above continuous to increase. Additionally, there has been an increase of transplants, mainly in those aged 50-64 and 65 and above[39]. Historically, due to the higher prevalence of comorbidities in this age group, there was an increased mortality from hepatic and nonhepatic causes[72], combined with a decreased likelihood of transplantation and consequently survival from older onset and stage of liver disease was historically poor. Nowadays, once transplanted, survival benefit differs little between groups with the same Model for End-Stage Liver Diseases (MELD) score[73].

Infection and malignancies remain the main causes of death after liver transplantation in older adults[73, 74]. The complexity of the surgical procedure will have a substantial impact on infectious complications[75]. Liver transplantation is often prolonged involving the need of large amounts of transfusion of blood products. Particular concerns include multi-drug-resistant organisms (MDROs) such as Enterobacteriaceae or non-albicans and echinocandin-resistant Candida spp. Bloodstream infections and sepsis are common in this population, often resulting from strictures resulting in cholangitis, or bile leaks with subsequent needs for drain or stent placements, or even re-operations. Of special note are mycobacterial infections as liver transplant recipients have an 18-fold increase in prevalence of active tuberculosis, with a 4-fold increase in mortality[76].

Viral hepatitides are especially important in liver transplantation and older adults[77]. The incidence of HCV in the younger population has increased although chronic HCV and its complications are more common in older adults. Age at time of HCV infection is associated with progression of disease[78]. Fortunately, tolerable and effective HCV treatments, even in extreme ages[79], are currently available and as such, the proportion of patients progressing to transplant with HCV has continued to decrease[39]. Furthermore, historically, many HCV organs were discarded, but these are now used to transplant into HCV positive as well as negative recipients with good results. The discussion of this topic is beyond the scope of this article. Regarding hepatitis B, older adults are generally at low risk for exposure. Similar acute hepatitis A and hepatitis E is less common in older adults but can result in high mortality[80].

### Lung transplantation

Patients aged 65 or above are the fastest growing group on the lung transplant waitlist and have the highest transplant rates per 100 waitlist years[37].

The incidence and prevalence of idiopathic pulmonary fibrosis (IPF) increases with age. Along with chronic obstructive pulmonary disease and bronchiectasis, these are the leading indication for lung transplantation in older adults. In combination with a change in the lung allocation system LAS, which prioritized risk of short-term mortality over time on the waitlist, as in IPF, has shifted lung transplantation towards older and sicker patients[81].

Historically, single orthotopic lung transplantation was preferred over bilateral orthotopic lung transplantation but this is an area of ongoing controversy. Bilateral lung transplantation offers a long term survival over single lung transplantation but also is associated with increased short-term complications. It seems that outcomes at age 75 and beyond are acceptable with single lung transplantation[82]. Older donors, who may be declined for younger potential recipients, have a lower overall and chronic lung allograft dysfunction (CLAD)-free survival[83], yet if centers take waiting list mortality into account, they may be valuable for older recipients.

The “increased” mortality of older patients between 1 month and 1 year after lung transplantation, seems to be predominantly from infectious causes and has been speculated to be secondary to immunosenescence of older adults[84]. In fact, the most frequent cause of death at one year after lung transplant in recipients aged 12 years or older are infections[37]. Type of infections vary depending on the underlying scenario. This can include pre-transplant colonization[85] with MDROs such as MDR Pseudomonas spp., *Mycobacterium abscessus* or *M. avium-intracellulare* among others, which can also result in intraoperative spillage with subsequent empyema in our personal experience. The lung allograft is in direct and continual exposure to the environment and therefore respiratory infections are frequent, especially in older adults. Mold infections are particularly frequent and early recognition is particularly important in infections due to non-Aspergillus spp. molds due to their high mortality[86]. Differentiating colonization from infection can be challenging.

Of special note in the lung transplant population are infections due to *Mycoplasma hominis* and *Ureaplasma urealyticum* which depend on urea hydrolysis to ammonia and carbon dioxide for energy production. Hyperammonemia is often the only clue to their presence[87] and these organisms are often not covered with the standard peri-transplant prophylaxis. There is not yet sufficient data to understand if these infections are more common or pathogenic in the older lung recipient.

### Other: intestinal and multivisceral transplantation, vascularized composite allotransplantation (VCA)

While these modalities of transplantation are associated with a high risk of infectious complications due to their underlying nature (intestinal, multivisceral) and need for profound immunosuppression (VCA), there is currently not much experience in the older patient.

## Immunosenescence and Immunosuppression

### Immunosenescence

The age related decline in immune function otherwise known as immunosenescence is a multifactorial concept with a variety of consequences[3]. It not only affects the innate and adaptive immune system but also alters the interface between innate and adaptive immune systems, and the ratio of memory and naïve T cells. This limits the ability of older adults to respond to pathogens. Chronic viral infections might alter the capacity of T cells to proliferate. CMV plays an important role as seroprevalence increases with age[88] its presence has been related to enhanced immunosenescence[89], and it is of critical importance for the risk stratification of transplant patients[90].

Inflamm-aging denotes the pro-inflammatory state that comes with aging[91]. Older adults encounter changes or barriers that will put them at increased risk of peri- and post-transplant infections. These are not only limited to alteration of barriers (e.g. skin, gastrointestinal), but also involve a decreased cough reflex, lack of immunization, loss of protective antibodies over time, or decreased antibody response to immunizations. The degree of exposure to certain pathogens and their types might differ in the setting of institutionalization, prolonged hospitalizations and use of catheters among others[89].

Immunosuppressive and other drug absorption can be a problem in patients with delayed gastric emptying, decreased splanchnic blood flow and changes in cytochrome isoenzymes[92, 93]. At this point in time there is limited data regarding immunosenescence in organ transplantation and vulnerability to infection[94, 95]. Yet, with much experimental data coming from outside of transplantation, its impact in this field is not completely understood[92, 96].

However, while chronological age is a risk factor for disease and mortality, the concept of biological aging denotes the heterogeneity of different biomarkers, genomic predictors and biological processes in individuals. While biologic aging is seen in many chronic diseases, we have little understanding of how these predictors work[97].

### Immunosuppression

To date, adjustments to the maintenance immunosuppression in older adults are infrequently made. The concept of individualized immunosuppression in this age group has been explored in kidney transplant recipients but has yet to be implemented routinely in every center[92, 98–100]. Although there has been a historical concern with use of thymoglobulin induction therapy in older patients, analysis of UNOS data demonstrated increased rates of rejection in high immunologic risk elderly kidney transplant recipients receiving high risk organs [101], others support that a lower dose of thymoglobulin might suffice[102]. Due to the increased immunogenicity of older organs, some authors suggest that recipients of these organs may require more potent early immunosuppression[96]. As described, older adults seem to have a high risk of infectious complications[6, 60] and potentially lower risk of rejection[103–105], thus further research in the area of optimal immunosuppression seems warranted.

## Post-transplant follow up

Post-transplant follow up for older adults should not differ from standard center-specific protocols. Some unique situations that are of increasing concern in this population are highlighted here.

### Vaccines

There is a distinct benefit of giving vaccinations pre-transplant, that is, pre-immunosuppression. Vaccination should occur pre-transplant whenever possible as per CDC and AST guidelines[106] but recognizing that immunosenescence results in older patients needing more frequent and extensive lists of recommended vaccines. This includes household and other close contacts to the patients. Live vaccines should be administered at least 4 weeks prior to immunosuppression and are generally contraindicated after SOT. For other post-transplant vaccinations, the optimal timing to resume immunizations has not been clearly defined but most centers will withheld them during at least the first 2 months after SOT[107]. Increasingly strong data supports safety of vaccines including certain adjuvant ones in the post-transplant phase without inducement of any alloreactivity or frank rejection[108]. The CDC has not made recommendations regarding the use of the recombinant zoster vaccine (RZV) in SOT[109, 110]. Preliminary data regarding the immunogenicity and safety of RZV in kidney transplant recipients after transplantation are promising[111] although long term follow-up and efficacy data are lacking.

### Skin cancers

Age by itself is a risk factor for skin cancer likely due to the accumulated exposure to ultraviolet radiation. In addition to this, SOT are approximately 65-250 times more likely to develop squamous cell carcinoma[112], which is more aggressive and associated with a higher mortality than in the non-immunosuppressed. Extensive counseling regarding this complication, sun protection, post-transplant surveillance and education regarding self-examination become important. Certain immunosuppressants such as CNI are associated with an increased risk of skin cancer when compared to mammalian target of rapamycin (mTOR) inhibitors[113]. Chemoprophylaxis to prevent skin cancer might be indicated in certain high-risk patients[114].

### Other malignancies following SOT

SOT recipients are at a higher risk of infection-related malignancies such as certain lymphoproliferative disorders (Epstein Barr Virus), anogenital and oropharyngeal cancers (Human Papillomavirus) and Kaposi sarcoma (Human Herpesvirus 8) as well as infection-unrelated cancers. Besides age and other individual risk factors (e.g. smoking history, genetics etc), CNI seem to be the principal risk factor for post-transplant malignancies[115, 116], while other agents such as mycophenolate mofetil and sirolimus have antitumor properties. Age appropriate cancer screening should be ensured prior to listing, and continued after SOT, as cancer-related mortality is higher than in the non-transplanted population[59].

### Increased cardiovascular complications

Age is associated with an increase in atherosclerosis. Transplant recipients frequently have several classic cardiovascular (CV) risk factors, in part exacerbated by the immunosuppression, e.g. prednisone and the risk of post-transplant diabetes mellitus. The one exception where CV risk is reduced after transplantation, yet still higher than in the general population, is in kidney transplant recipients. Aggressive management of comorbidities and CV risk factors is encouraged[117].

### Osteoporosis

Increasing age is an independent risk factor for osteoporosis and postoperative osteoporotic fractures[118]. Immunosuppression, such as prednisone, contribute to further bone loss. In addition, vitamin D levels are often low in the transplant population. Increased screening, vitamin supplementation and biphosponates might be necessary.

## Conclusions

Guidelines for the assessment and follow up of older SOT candidates and recipients are poorly defined and for the most part lacking. However, what data does exists suggests outcomes after transplant can be very good. Often, this is a carefully selected patient population. Transplant care providers should be familiar with some important geriatric terms, and optimizing selection of older adults undergoing SOT should be considered. Infections are frequent after SOT, especially in older populations. Despite these complications and even with the use of suboptimal donors, overall outcomes are favorable. Post-transplant care in older adults should be optimized; especially immunosuppressive and antimicrobial prophylactic protocols might need adjustment. The absence of standardized measurements in this patient population is a major gap and opportunity for future research.
